# Maternal mortality in Bangladesh: Who, when, why, and where? A national survey-based analysis

**DOI:** 10.7189/jogh.13.07002

**Published:** 2023-06-09

**Authors:** Aniqa Tasnim Hossain, Abu Bakkar Siddique, Sabrina Jabeen, Shusmita Khan, M Moinuddin Haider, Shafiqul Ameen, Tazeen Tahsina, Nitai Chakraborty, Quamrun Nahar, Kanta Jamil, Shams El Arifeen, Ahmed Ehsanur Rahman

**Affiliations:** 1 International Centre for Diarrhoeal Disease Research, Bangladesh; 2 Data for Impact, University of North Carolina at Chapel Hill, Chapel Hill, USA; 3 Independent consultant

## Abstract

**Background:**

Despite a notable decline in recent decades, maternal mortality in Bangladesh remains high. A thorough understanding of causes of maternal deaths is essential for effective policy and programme planning. Here we report the current level and major causes of maternal deaths in Bangladesh, focusing on care-seeking practices, timing, and place of deaths.

**Methods:**

We analysed data from the 2016 Bangladesh Maternal Mortality and Health Care Survey (BMMS), conducted with nationally representative sample of 298 284 households. We adapted the World Health Organization’s 2014 verbal autopsy (VA) questionnaire. Trained physicians reviewed the responses and assigned the cause of death based on the International Classification of Diseases (ICD-10). We included 175 maternal deaths in our analysis.

**Results:**

The maternal mortality ratio was 196 (uncertainty range = 159-234) per 100 000 live births. Thirty-eight per cent of maternal deaths occurred on the day of delivery and 6% on one day post-delivery. Nineteen per cent of the maternal deaths occurred at home, another 19% in-transit, almost half (49%) in a public facility, and 13% in a private hospital. Haemorrhage contributed to 31% and eclampsia to 23% of the maternal deaths. Twenty-one per cent of the maternal deaths occurred due to indirect causes. Ninety-two per cent sought care before dying, of which 7% sought care from home. Thirty-three per cent of women who died due to maternal causes sought care from three or more different places, indicating they were substantially shuttled between facilities. Eighty per cent of the deceased women who delivered in a public facility also died in a public facility.

**Conclusions:**

Two major causes accounted for around half of all maternal deaths, and almost half occurred during childbirth and by two days of birth. Interventions to address these two causes should be prioritised to improve the provision and experience of care during childbirth. Significant investments are required for facilitating emergency transportation and ensuring accountability in the overall referral practices.

Over the past decades, Bangladesh experienced remarkable growth in many health and population indicators, including one of the fastest reductions in maternal mortality among low- and middle-income countries (LMICs) [1,2]. These declines were especially remarkable considering the constraints faced by health systems and other contextual factors. The Sustainable Development Goals (SDGs) aim to reduce the maternal mortality ratio (MMR) to less than 70 per 100 000 live births by 2030 [3-6], requiring Bangladesh to double the Millenium Development Goal (MDG) era annual rate of reduction.

Maternal deaths refer to the death of a woman while pregnant or within 42 days of end of pregnancy, regardless of the duration and place of the pregnancy, from any cause related to or worsened by the pregnancy or its management (excluding accidental or incidental causes) [7-9]. Updated estimates on the causes of maternal deaths are crucial for reinvigorating strategies and interventions for maternal health decisions. Moreover, unpacking information on the timing and places for different causes of maternal deaths is crucial to reducing maternal mortality, as it will assist in appropriate resource allocation and health systems planning.

Unfortunately, effective civil registration and vital statistics systems for providing accurate information on maternal deaths are either incomplete or unavailable in most of LMICs, including Bangladesh [10,11]. The 2016 Bangladesh Maternal Mortality and Health Care Survey (BMMS) is the third nationally representative survey that was conducted to understand the country’s progress toward global and national targets on maternal health and estimate the major causes of maternal deaths [12]. Here we present the current level and major causes of maternal deaths in Bangladesh, focusing on care-seeking practices, timing, and place of death.

## METHODS

We conducted a secondary analysis of the third round of the BMMS conducted in 2016, which used nationally representative sample with a multi-stage sampling procedure to measure the MMR with a three-year recall period [12]. The BMMS was conducted by the National Institute of Population Research and Training (NIPORT) of the Government of Bangladesh, with technical assistance from the International Centre for Diarrhoeal Disease Research, Bangladesh (icddr,b) and the Monitoring and Evaluation to Assess and Use Results (MEASURE) programme, and with financial support from the United States Agency for International Development (USAID).

The 2016 BMMS was conducted among 298 284 households. Trained enumerators visited the households and administered a structured questionnaire to collect information on household members and selected indicators. The household questionnaire had a section to list all deaths among the usual residents of the surveyed households. If any death was reported, additional information on sex, age at death, and time of death was collected. For the deceased women (13-49 years of age), four questions were asked as to whether the woman died while she was pregnant, giving birth, within 42 days, or after 42 days to one year following the end of the pregnancy. A separate group of trained interviewers used a verbal autopsy (VA) questionnaire, adapted from the WHO’s 2016 standard VA instrument [13] and translated into Bangla, to conduct follow-up structured interviews with a household member who knew the most about the deceased person. VA interviews were conducted for all deaths reported among women aged 13-49 years at the time of death, but the analysis presented here is limited to women aged 15-49 years.

Two physicians reviewed each questionnaire after receiving intensive 21-day training (including 14 days of practice using the previous rounds of BMMS VA questionnaire) from master trainers previously involved with other national surveys using VA and International Classification of Diseases (ICD) codes. Each of the two physicians assigned a final cause of death (direct, underlying, and contributory). If disagreements occurred, they were resolved by a third physician. An expert obstetrician committee was also involved in assigning a specific cause of maternal death when the three physicians agreed that the death was maternal, but could not assign a specific cause and recorded it as “undetermined”. The results presented here represent the underlying causes of deaths. The additional details on methodology and cause of death assignments are available in the BMMS reports [12,14,15].

### Analysis plan

We used Stata software (version 14; StataCorp LLC, USA) for data analysis [16]. We used descriptive statistics to report the level, trends, and patterns of the MMR with a 95% confidence interval (CI) based on the 2016 BMMS. We used 2016 BMMS data to present the cause-specific mortality fraction and cause-specific mortality ratio. We grouped similar ICD-10 codes and presented the causes of death in seven broad categories (Table S1 in the **Online Supplementary Document**). We used the total population reported in the 2011 census report and average annual growth rate of Bangladesh reported by the World Bank to project the population in 2016 [17]. Based on this population, we used the crude birth rate in the 2016 BMMS report to estimate the number of live births in 2016. We then calculated the number of cause-specific maternal deaths based on the cause-specific mortality ratio (per 100 000 live births) in 2016. The uncertainty range (UR) was calculated using the CIs obtained for the cause-specific mortality ratios.

We categorised the overall timing by three different periods of childbirth: during pregnancy, after the start of labour pains but before birth, and after delivery. We further disaggregated and presented timing of death into three broad categories: during pregnancy (first trimester, second trimester, and third trimester); during delivery (after labour pains but before birth on day 0); and after delivery (day 0, 1 day after, 2 days after, 2-6 days after, 7-42 days after, and ≥43 days after delivery). We categorised place of death as home, facility (public or private), and during transit. We also presented the care-seeking patterns using descriptive statistics. Last, we reviewed the open-ended questions in the VA forms and conducted narrative synthesis to have a deeper understanding about the causes of maternal deaths.

### Ethical approval

We used publicly available data and obtained approval from NIPORT and MEASURE Evaluation for this analysis.

## RESULTS

The BMMS 2016 surveyed 298 284 households, from which 321 214 women aged 13-49 years were identified. From these interviews, we identified 1524 females deaths (13-49 years), of which 175 maternal deaths (15-49 years) according to WHO definition.

**Figure 1:**
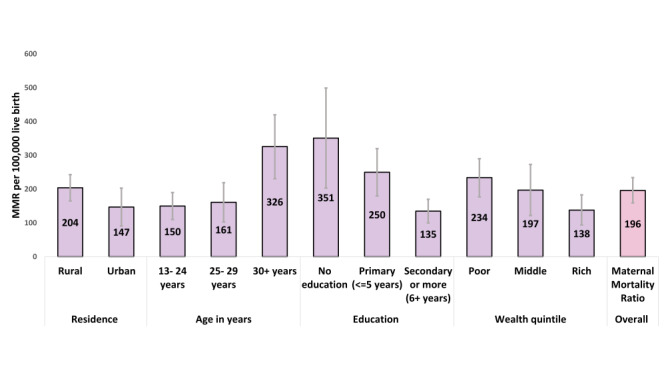
MMR by background characteristics, presented in deaths per 100 000 live births.

**Figure 1** and Table S2 in the **Online Supplementary Document** show estimates of the overall MMR disaggregated by residence, age of the women, education, and wealth status. The overall MMR was 196 (UR = 159-234) per 100 000 live births. The MMR was higher among women with no education (351 per 100 000 live births; UR = 203-499) than those with secondary or higher level of education (135 per 100 000 live births; UR 100-170). MMR was 204 (UR = 165-243) in rural areas and 147 (UR = 91-203) in urban areas. Women in the poor wealth category had an MMR of 234 (UR = 177-290) compared with the women in the rich wealth category, at 138 (UR = 94-183). Figures S1 and S2 in the **Online Supplementary Document** show the equity gaps in MMR by background characteristics over years 2001, 2010, and 2016. The gap in MMR between rural and urban areas increased in 2016 compared with 2001 and 2010. The gap in MMR between the poorer and richer families remained the same across these three survey years.

**Figure 2:**
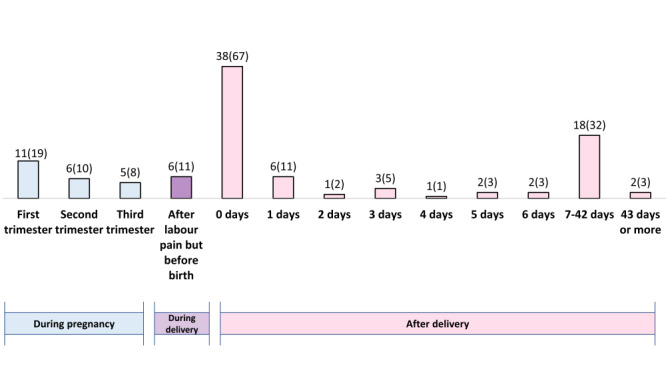
Timing of maternal deaths, presented in percentages and numbers (% (n)) (n = 175).

**Figure 2** shows the percentage distributions and number of maternal deaths across antepartum, peripartum and postpartum periods in Bangladesh. Overall, almost half of maternal deaths happened during delivery (6%), on the day of the delivery (38%), and the first day after delivery (6%).

**Figure 3:**
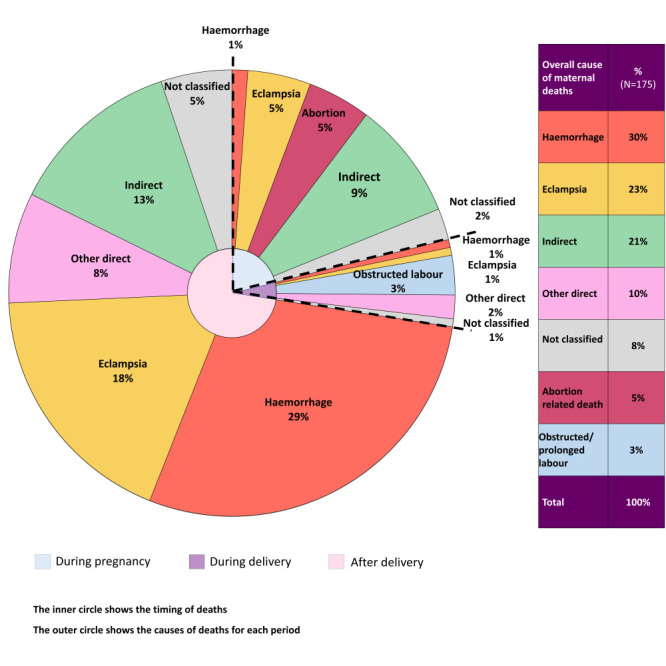
Causes of maternal death by timing of death, presented in percentages (n = 175).

**Figure 3** presents the percentage distributions of the causes of maternal death by timing of death. The two main causes were haemorrhage (30%) and eclampsia (23%). Twenty-one per cent of maternal deaths were associated with other indirect causes. Five per cent were due to abortion-related causes and 3% occurred due to obstructed or prolonged labour.

**Figure 4:**
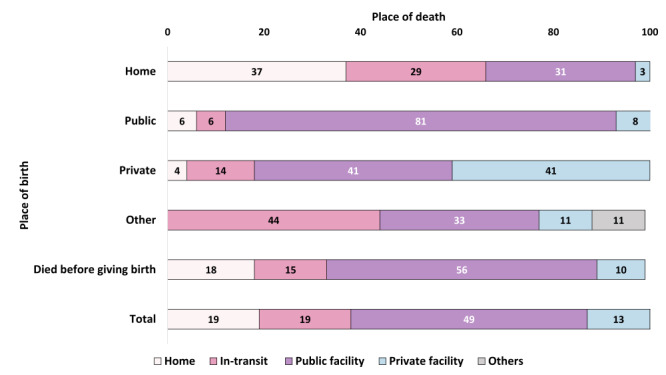
Place of maternal deaths by place of birth, presented in percentages (n = 175).

**Figure 4** presents the place of maternal deaths by place of birth. Overall, 19% died at home, 19% died in-transit, almost half (49%) in a public facility, and 13% died in private facilities. Thirty-seven per cent of the women who gave birth at home died at home, 29% died in-transit, 31% died in a public facility, and 3% died in a private facility. Eighty-one per cent of the women who gave birth in a public facility also died in one. Among the women who gave birth in a private facility, 41% died in a public facility and 41% died in a private facility. A smaller proportion (14%) of these women died at home or in-transit.

**Figure 5:**
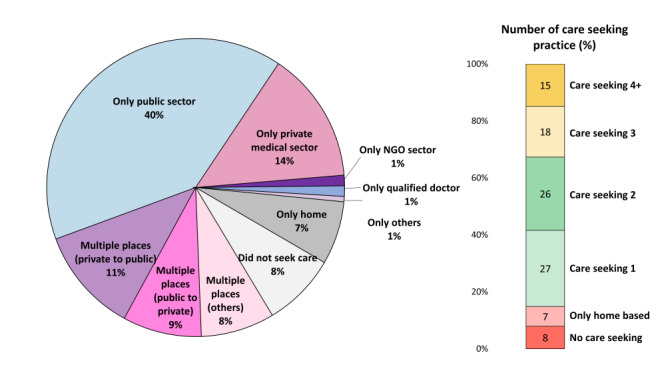
Care-seeking practices of the deceased women by type of provider, presented in percentages (n = 175).

**Figure 5** and Figure S3 in the **Online Supplementary Document** present the proportions of care-seeking practices by type of providers, which include government, private, and nongovernmental organization sectors. Ninety-two per cent of the women who died due to maternal causes sought care before death, of whom 85% sought care outside home. Forty per cent of these women sought care from public facilities only, and 14% of sought care from private facilities only. Twenty-eight per cent sought care from multiple places; 15% sought care four or more times and 18% sought care three times before their deaths. Nine per cent sought care initially from a public sector facility and then moved to the private sector, and 11% sought care initially from the private sector and then shifted to a public sector facility.

## DISCUSSION

Bangladesh has made significant progress in health outcomes over the past few decades [18]. We observed a significant reduction in maternal mortality ratio from 2001 to 2010, with an average annual rate of reduction of 5.1%. However, the MMR showed an apparent stalling between 2010 and 2016, highlighting gaps in prioritisation of interventions and health systems planning for maternal health and reinforcing the need for redesigning existing strategies [19]. Here we address a key evidence gap related to the current level and key causes of maternal deaths in Bangladesh, focusing on care-seeking practices and timing and place of deaths. We observed that women living in rural areas and those of poorer socioeconomic status were more likely to die due to maternal causes of deaths. Most deaths occurred on the day of and one day after delivery. We identified haemorrhage and eclampsia as the major contributors to maternal deaths. We also found a large proportion of deaths occurring in-transit with substantial shuttling for care seeking before their deaths.

The initial reduction in overall maternal mortality between 2001 and 2010 can be explained by an early enthusiasm of the Bangladeshi Government in implementing strategies and programmes at the beginning of the MDG era. The programmes included (among other things) emergency obstetric and newborn care training, community-based skilled birth attendant training, a demand-side financing program, and the maternal and perinatal death surveillance and response (MPDSR) initiative. Although these interventions and programmes helped achieve the easier targets concerning mortality reductions, further ones remained unaccomplished.

We found that the MMR was higher in rural areas and among poor older women. Strengthening appropriate quality emergency obstetric care in rural areas has been a major challenge [20]. One explanation for the higher MMR in rural areas could be the disparities in rural and urban health systems. The better health systems and prevalent private healthcare sector facilities in urban areas offer more opportunities for achieving better access to care, while rural areas are more public sector-dependent, limiting access to care. Also, the proportion of home deliveries is higher in rural Bangladesh (57%) than in urban areas (40%) [12]. Moreover, 51% of the deliveries were conducted in rural areas with the help of a traditional birth attendant or unqualified providers, whereas in urban areas, this proportion was lower at 31% [21]. This may contribute to lowering the probability of safe birth in rural area.

We also noted that poorer women had higher maternal mortality than wealthier ones. WHO has reported similar findings in its factsheet on maternal mortality [22]. Receipt of quality antenatal care (ANC) is associated with wealth status. Only 7% of women from the lowest wealth quintile were observed to have received quality ANC, compared with 37% of women from the highest wealth quintile [21]. Moreover, the percentage of women who delivered at a health facility in the lowest wealth quintile was only 26%, whereas it was 78% among women in the highest wealth quintile [21]. Such inequities may explain the differences in MMR across the wealth categories.

Regarding the timing of maternal deaths, we observed that half occurred during birth and immediately after delivery. This is in line with findings of a global study by Kassebaum et al. [23] who reported that around 25% of maternal deaths occurred in the antepartum period (before the onset of labour); another 25% in the intrapartum and immediate postpartum periods (up to 24 hours after delivery), one-third in the delayed postpartum periods (24 hours to 42 days after delivery), and 12% in the late postpartum period (43 days to 1 year after delivery). Another study conducted in Pakistan also reported similar findings. We found that two-thirds of maternal deaths occurred in the postpartum period and almost half within 24 hours of delivery [24]. However, in Bangladesh, facilities are not ready for the appropriate and timely management of maternal complications where most births take place. WHO recommends 13 items to assess facility readiness for normal vaginal delivery. Overall, less than 1% of facilities have all the 13 items to provide normal delivery services in Bangladesh [25]. Signal functions can play a role in emergency management of maternal complications, but only 5% of Bangladeshi facilities performed all comprehensive emergency obstetric and neonatal care signal functions in past three months, which indicates the gross level non-functionality for emergency maternal management [25]. A focus should be placed on the availability and readiness of interventions during childbirth and immediately after birth to avert the preventable maternal deaths.

Around one-fifth of the maternal deaths in our study took place in-transit, coinciding with the findings from a study conducted in India [26]which reported that 18% of maternal deaths occurred in-transit and one-tenth died while being referred between health facilities. Lack of emergency transportation and appropriate referral may have primarily caused this in-transit deaths in Bangladesh. Only 30% of the healthcare facilities offering normal delivery services have a functioning ambulance or other vehicles for emergency transport [25]. There is also no structured system of emergency medical transportation from communities to facilities [27,28]. Moreover, delay in recognition of complication and decision-making for care-seeking were also among contributing factors. Narrative synthesis of these deaths reveals that two-thirds of the women who died in-transit experienced a delay in the identification of the specific health issue, four-fifths had a delay in decision-making, and approximately half sought care multiple times before death. The other possible explanation was the gaps in birth-preparedness particularly related to arranging emergency transportation [29]. One of the possible ways we can ensure birth preparedness is by providing quality antenatal care. However, there are gaps in provision of quality ANC with appropriate counselling in Bangladesh [30]. A national community survey reported only 18% of women received quality ANC and only 40% of mothers who received ANC reported that providers informed them of the signs of possible pregnancy complications during the visit [21]. Another community-survey based study reported healthcare providers counselled women on danger signs in only two-third of the ANC contacts [31]. Thus, ANC with appropriate counselling has to be ensured for birth-preparedness and complication readiness. Moreover, a structured referral pathway between healthcare facilities and emergency transportation also need to be ensured to avert in-transit deaths.

We also observed that most deceased mothers sought care before death, but were also substantially shuttled between facilities, which is concerning. A lack of structured referral, absence of emergency transportation and delay in decision-making, and appropriate care-seeking can potentially explain this shuttling and in-transit deaths, as can a lack of accountability of the private sectors. We observed more women die in public facilities when they gave birth in public facilities, but lower proportion of maternal deaths occur in private facilities when they gave birth in private facilities. The national facility survey showed less than 1% private facilities had readiness to provide normal delivery services. This indicates, the private facilities lack quality to manage complications and are prone to inappropriate referral to public facility without necessary stabilisation and facilitation. The policies should be prioritised for strengthening accountability of the private facilities to avert shuttling.

We identified haemorrhage as the most common cause of maternal death, causing more than 2000 deaths in Bangladesh (Figure S4 in the **Online Supplementary Document**). Approximately 35% of maternal deaths in LMICs are due to haemorrhage, with some variations across countries [32]. Postpartum haemorrhage (PPH), which refers to the loss of 500 ml or more of blood, is responsible for most of the haemmorhage-related maternal deaths [33]. Evidence suggests that 88% of women who die from PPH die within four hours of delivery [34]. This high rate of obstetric haemorrhagic deaths may be explained by the lack of appropriate birth preparedness and complication readiness, quality ANC to identify high-risk pregnancies, and facility readiness to manage haemorrhage-related complications [21,35]. For the prevention of PPH, all women giving birth should be offered uterotonics during the third stage of labour. While oxytocin is recommended, other injectable uterotonics and misoprostol are recommended as alternatives for the prevention of PPH in settings where oxytocin is unavailable [36,37]. In Bangladesh, only 57% of facilities had misoprostol capsules or tablets as reported in the recent national facility survey [25]. Importantly, blood transfusion is a major component for emergency management of PPH [38], yet only 4% of the facilities reported that they provide blood transfusion services in Bangladesh [25], which may explain the high rate of haemorrhagic deaths in Bangladesh.

Wealso found that eclampsia was the second largest cause of maternal death, causing nearly 1600 deaths in the country (Figure S4 in the **Online Supplementary Document**). Eclampsia remains an important cause of maternal mortality throughout the world, accounting for about 50 000 deaths globally [39]. LMICs often confront this health hazard because of illiteracy, lack of health awareness and education, poverty, and superstitious beliefs that prohibit women from seeking appropriate medical advice during pregnancy [40]. The fundamentals of eclampsia management include control of convulsions, control of severe hypertension, the initiation of steps to effect delivery, and general nursing care [40,41]. The remedy against eclampsia varies across different countries. Bangladesh participated in the recent Magpie trial, which showed that magnesium sulphate can reduce the risk of eclampsia among women [42-44]. However, injectable magnesium sulphate for management of eclampsia is available in only 14% of facilities in Bangladesh. It is also indispensable to identify the high-risk pre-eclampsia patients by providing appropriate ANC with blood pressure and urine protein measurements. However, the health facility survey suggests less than half of the facilities had urine protein testing capacity. Ensuring quality ANC and appropriate prevention and treatment strategy could decrease the potential hazards of eclampsia.

The third largest cause of maternal cause – indirect causes – include heart, liver, lung, or brain dysfunctions, contributing 21% of the maternal deaths in Bangladesh (Figure S4 in the **Online Supplementary Document**) and 27% globally [45]. South Asia and sub-Saharan Africa have the largest share of these deaths in the global context [45,46]. Globally, we observe a growing direct and indirect effect of non-communicable diseases on maternal mortality [23]. There are significant gaps in addressing these indirect causes of death despite their observed severity [47], necessitating initiatives to comprehensively understand these causes of deaths and develop appropriate responses.

Maternal deaths should be identified and reported in real time. Every death should be assessed and its medical causes and related health systems gaps identified, providing information for customising the management approach and strengthening health systems. For materialising this vision, Bangladesh had been implementing Maternal and Perinatal Death Surveillance and Response (MPDRS) since 2010 [48]; this initiative involving midwives was envisioned as one of the key approaches for quality improvement in maternal and newborn health, by measuring maternal and perinatal mortality in real time involving the community [49,50]. Currently, MPDSR is covering 48 districts and over two third of population of Bangladesh [48]. Despite achieving sub-national coverage, this has not helped in averting these maternal deaths substantially, since in Bangladesh, the initiative is primarily focused on reviewing maternal deaths. There are major delays in analysis and reporting with almost non-existent response strategy, which are fundamental components of MPDSR. Significant investment should be made to strengthen the capacity of analysis and emphasise on response strategy at a national level.

### Strengths and limitations

We analysed the major causes of maternal death presented here using the most recently published nationally representative 2016 BMMS [12] based on VA surveys. These surveys can provide information on the duration of labour, progress of labour, excessive bleeding, duration of bleeding, initial management, care-seeking behaviour for different causes of deaths, and their appropriate management. We included representative samples across different populations’ sociodemographic characteristics and all administrative divisions of Bangladesh, covering both urban and rural areas, which ensures the generalisability of the results. We used the validated WHO standard VA tool for data collection, pre-testing for its validity in Bangladesh. The physicians received comprehensive training on assigning the causes of death based on the ICD-10 codes. The authors were part of the training team for the data collectors and cause of death reviewers and ensured the quality of data collection during field work, which confirms the reliability of our results.

Despite having a nationally representative sample, we acknowledge that the sample size was not sufficient to provide disaggregated estimates by different background characteristics or care-seeking practices with an acceptable level of precision. Recall error and bias may have occurred because the data were collected retrospectively from close family members of the deceased women. The clinical questions asked during the interview could have been intimidating for the respondent to answer appropriately.

We also acknowledge that the physicians’ interpretations of data recorded in the questionnaires involves subjectivity, judgment, and personal bias. For some cases, the assignment of causes of death could have been misclassified. Nonetheless, the reviewers underwent rigorous training before the cause of death assignment; thus, subject-specific misclassification issues were likely minimised.

## CONCLUSIONS

Haemorrhage and eclampsia were the major maternal killers accounting for around half of the maternal deaths, almost half of which occurred on the day of and one day after delivery after substantial shuttling. Prioritisation of interventions focusing on these two causes and timing of deaths is necessary to avert the maternal deaths. We also recommend investing in the development of a structured referral system and emergency transportation to prevent the in-transit deaths. Establishing an accountability mechanism for private facilities focusing on complication management is important for reducing the shuttling of care-seeking before death. Lastly, an appropriate response strategy in addition to rapid identification of the deaths can support policymakers in making timely decisions to improve maternal health outcomes and avert future maternal deaths.

## Additional material


Online Supplementary Document
