# Supplementary material for: Maternal mortality in Bangladesh: Who, when, why, and where? A national survey-based analysis
Source: J Glob Health. 2023 Jun 9;13:07002. doi: 10.7189/jogh.13.07002 (PMC10248997; doi:10.7189/jogh.13.07002)
Supplement: Online Supplementary Document [file jogh-13-07002-s001.pdf]

Figure S1: Trends in MMR per 100,000 live births in Bangladesh with 95% Confidence Interval

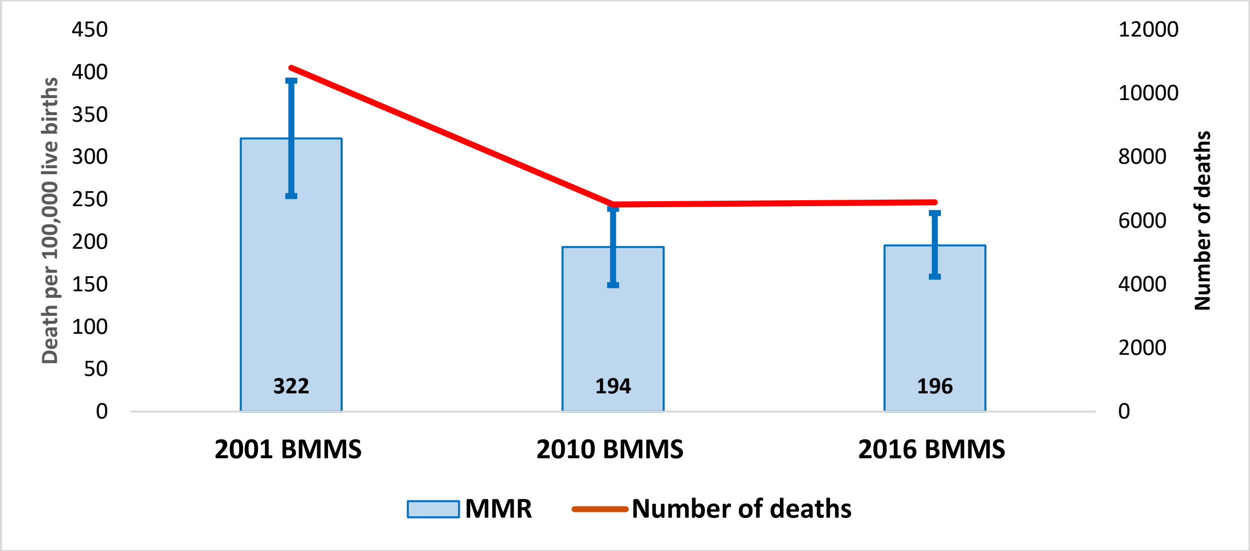

Figure S2: MMR by background characteristics in Bangladesh in survey years 2001, 2010, and 2016, presented in deaths per 100,000 live births

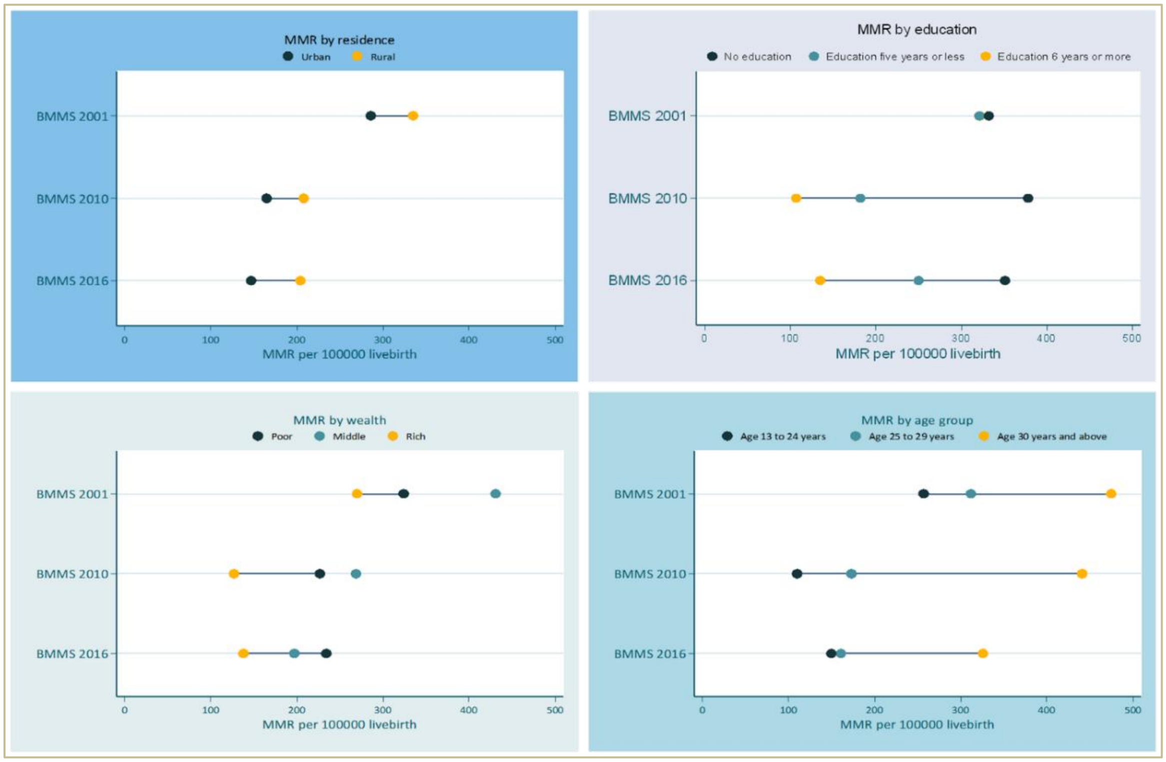

Figure S3: Flowchart of care-seeking practices among women of reproductive age (15–49 years) in Bangladesh, presented in percentages and numbers (n=175)

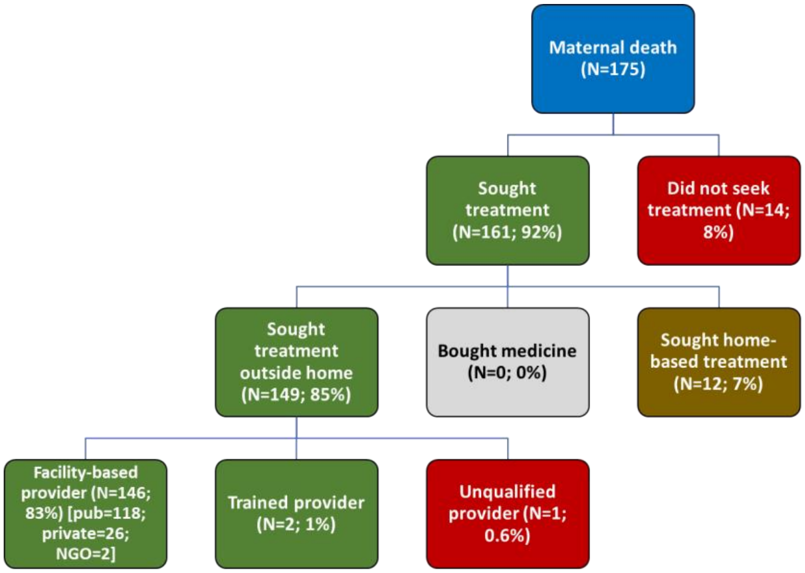

Figure S4: Estimated number of maternal deaths in 2016 by cause of maternal death in Bangladesh, presented in numbers

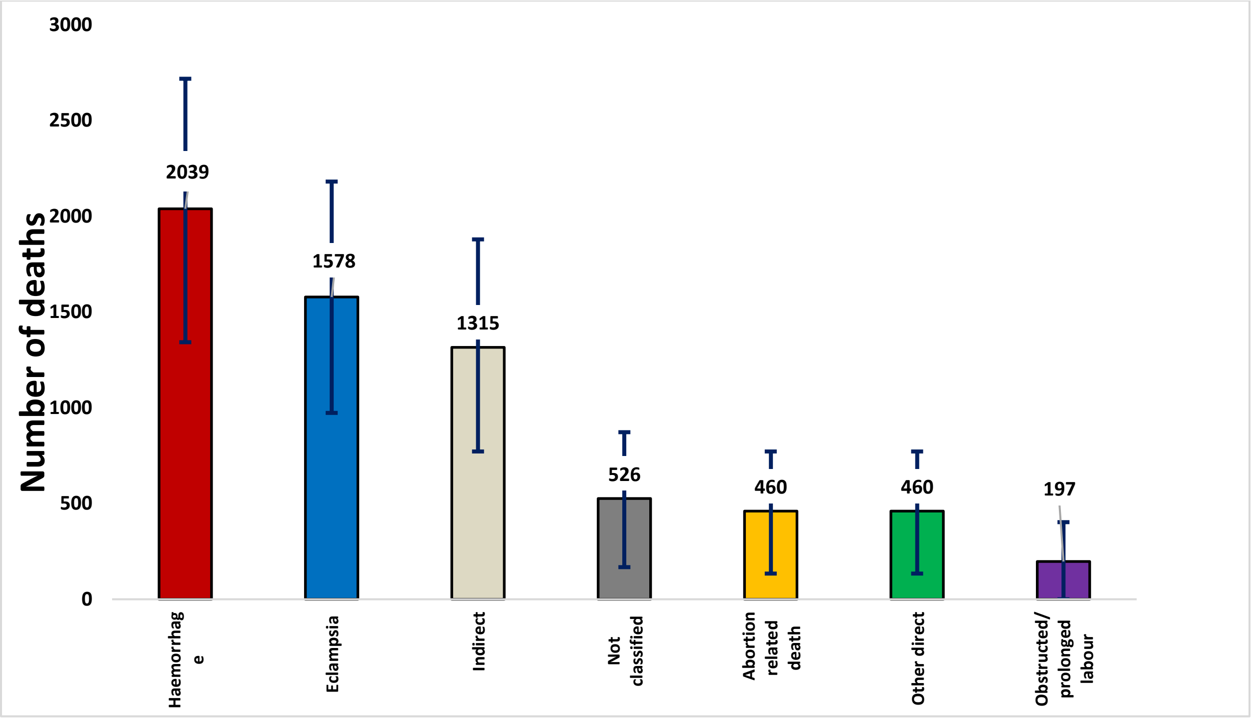

Table S1: Summary matrix of ICD-10 codes used to present the cause of death in categories

ICD-10 Code, 2010 edition	Title
O00	Ectopic pregnancy
O00.0	Abdominal pregnancy
O00.1	Tubal pregnancy
O00.2	Ovarian pregnancy
O00.8	Other ectopic pregnancy
O00.9	Ectopic pregnancy, unspecified
O01	Hydatidiform mole
O01.0	Complete hydatidiform mole
O01.1	Incomplete and partial hydatidiform mole
O01.9	Hydatidiform mole, unspecified
O02	Other abnormal products of conception
O02.0	Blighted ovum and nonhydatidiform mole
O02.1	Missed abortion
O02.8	Other specified abnormal products of conception
O02.9	Abnormal product of conception, unspecified
O03	Spontaneous abortion
O03.0	Spontaneous abortion : incomplete, complicated by genital tract and pelvic infection
O03.1	Spontaneous abortion : incomplete, complicated by delayed or excessive haemorrhage
O03.2	Spontaneous abortion : incomplete, complicated by embolism
O03.3	Spontaneous abortion : incomplete, with other and unspecified complications
O03.4	Spontaneous abortion : incomplete, without complication
O03.5	Spontaneous abortion : complete or unspecified, complicated by genital tract and pelvic infection
O03.6	Spontaneous abortion : complete or unspecified, complicated by delayed or excessive haemorrhage
O03.7	Spontaneous abortion : complete or unspecified, complicated by embolism
O03.8	Spontaneous abortion : complete or unspecified, with other and unspecified complications
O03.9	Spontaneous abortion : complete or unspecified, without complication
O04	Induced abortion
O04.0	Medical abortion : incomplete, complicated by genital tract and pelvic infection
O04.1	Medical abortion : incomplete, complicated by delayed or excessive haemorrhage
O04.2	Medical abortion : incomplete, complicated by embolism
O04.3	Medical abortion : incomplete, with other and unspecified complications
O04.4	Medical abortion : incomplete, without complication
O04.5	Medical abortion : complete or unspecified, complicated by genital tract and pelvic infection
O04.6	Medical abortion : complete or unspecified, complicated by delayed or excessive haemorrhage
O04.7	Medical abortion : complete or unspecified, complicated by embolism
O04.8	Medical abortion : complete or unspecified, with other and unspecified complications
O04.9	Medical abortion : complete or unspecified, without complication
O05	Complications following abortion and ectopic and molar pregnancy
O05.0	Other abortion : incomplete, complicated by genital tract and pelvic infection
O05.1	Other abortion : incomplete, complicated by delayed or excessive haemorrhage
O05.2	Other abortion : incomplete, complicated by embolism
O05.3	Other abortion : incomplete, with other and unspecified complications
O05.4	Other abortion : incomplete, without complication
O05.5	Other abortion : complete or unspecified, complicated by genital tract and pelvic infection
O05.6	Other abortion : complete or unspecified, complicated by delayed or excessive haemorrhage
O05.7	Other abortion : complete or unspecified, complicated by embolism
O05.8	Other abortion : complete or unspecified, with other and unspecified complications
O05.9	Other abortion : complete or unspecified, without complication
O06	Unspecified abortion
O06.0	Unspecified abortion : incomplete, complicated by genital tract and pelvic infection
O06.1	Unspecified abortion : incomplete, complicated by delayed or excessive haemorrhage
O06.2	Unspecified abortion : incomplete, complicated by embolism
O06.3	Unspecified abortion : incomplete, with other and unspecified complications
O06.4	Unspecified abortion : incomplete, without complication

O06.5	Unspecified abortion : complete or unspecified, complicated by genital tract and pelvic infection
O06.6	Unspecified abortion : complete or unspecified, complicated by delayed or excessive haemorrhage
O06.7	Unspecified abortion : complete or unspecified, complicated by embolism
O06.8	Unspecified abortion : complete or unspecified, with other and unspecified complications
O06.9	Unspecified abortion : complete or unspecified, without complication
O07	Failed attempted abortion
O07.0	Failed medical abortion, complicated by genital tract and pelvic infection
O07.1	Failed medical abortion, complicated by delayed or excessive haemorrhage
O07.2	Failed medical abortion, complicated by embolism
O07.3	Failed medical abortion, with other and unspecified complications
O07.4	Failed medical abortion, without complication
O07.5	Other and unspecified failed attempted abortion, complicated by genital tract and pelvic infection
O07.6	Other and unspecified failed attempted abortion, complicated by delayed or excessive haemorrhage
O07.7	Other and unspecified failed attempted abortion, complicated by embolism
O07.8	Other and unspecified failed attempted abortion, with other and unspecified complications
O07.9	Other and unspecified failed attempted abortion, without complication
O11-O16	Oedema, proteinuria and hypertensive disorders in pregnancy, childbirth and the puerperium
O11	Pre-eclampsia superimposed on chronic hypertension
O12	Gestational [pregnancy-induced] oedema and proteinuria without hypertension
O12.0	Gestational oedema
O12.1	Gestational proteinuria
O12.2	Gestational oedema with proteinuria
O13	Gestational [pregnancy-induced] hypertension
O14	Pre-eclampsia
O14.0	Mild pre-eclampsia
O14.1	Severe pre-eclampsia
**O14.2**	HELLP syndrome
O14.9	Pre-eclampsia, unspecified
O15	Eclampsia
O15.0	Eclampsia in pregnancy
O15.1	Eclampsia in labour
O15.2	Eclampsia in the puerperium
O15.9	Eclampsia, unspecified as to time period
O16	Unspecified maternal hypertension
O20	Haemorrhage in early pregnancy
O20.0	Threatened abortion
O20.8	Other haemorrhage in early pregnancy
O20.9	Haemorrhage in early pregnancy, unspecified
O43	Placental disorders
O43.2	Morbidly adherent placenta
O44	Placenta praevia
O44.1	Placenta praevia with haemorrhage
O45	Premature separation of placenta [abruptio placentae]
O45.0	Premature separation of placenta with coagulation defect
O45.8	Other premature separation of placenta
O45.9	Premature separation of placenta, unspecified
O46	Antepartum haemorrhage, not elsewhere classified
O46.0	Antepartum haemorrhage with coagulation defect
O46.8	Other antepartum haemorrhage
O46.9	Antepartum haemorrhage, unspecified
O67	Labour and delivery complicated by intrapartum haemorrhage, not elsewhere classified
O67.0	Intrapartum haemorrhage with coagulation defect
O67.8	Other intrapartum haemorrhage
O67.9	Intrapartum haemorrhage, unspecified
O71.0	Rupture of uterus before onset of labour
O71.1	Rupture of uterus during labour
O71.3	Obstetric laceration of cervix
O71.4	Obstetric high vaginal laceration alone
O71.7	Obstetric haematoma of pelvis
O72	Postpartum haemorrhage
O72.0	Third-stage haemorrhage
O72.1	Other immediate postpartum haemorrhage
O72.2	Delayed and secondary postpartum haemorrhage

O72.3	Postpartum coagulation defects
O23	Infections of genitourinary tract in pregnancy
O23.0	Infections of kidney in pregnancy
O23.1	Infections of bladder in pregnancy
O23.2	Infections of urethra in pregnancy
O23.3	Infections of other parts of urinary tract in pregnancy
O23.4	Unspecified infection of urinary tract in pregnancy
O23.5	Infections of the genital tract in pregnancy
O23.9	Other and unspecified genitourinary tract infection in pregnancy
O41.1	Infection of amniotic sac and membranes
O75.3	Other infection during labour
O85	Puerperal sepsis
O86	Other puerperal infections
O86.0	Infection of obstetric surgical wound
O86.1	Other infection of genital tract following delivery
O86.2	Urinary tract infection following delivery
O86.3	Other genitourinary tract infections following delivery
O86.4	Pyrexia of unknown origin following delivery
O86.8	Other specified puerperal infections
O91	Infections of breast associated with childbirth
O91.0	Infection of nipple associated with childbirth
O91.1	Abscess of breast associated with childbirth
O91.2	Nonpurulent mastitis associated with childbirth
A34	Obstetrical tetanus
O21.1	Hyperemesis gravidarum with metabolic disturbance
O21.2	Late vomiting of pregnancy
O22	Venous complications in pregnancy
O22.3	Deep phlebothrombosis in pregnancy
O22.5	Cerebral venous thrombosis in pregnancy
O22.8	Other venous complications in pregnancy
O22.9	Venous complication in pregnancy, unspecified
O24.4	Diabetes mellitus arising in pregnancy
O26.6	Liver disorders in pregnancy, childbirth and the puerperium
O44.0	Placenta praevia specified as without haemorrhage
O71.2	Postpartum inversion of uterus
O71.5	Other obstetric injury to pelvic organs
O71.6	Obstetric damage to pelvic joints and ligaments
O71.8	Other specified obstetric trauma
O71.9	Obstetric trauma, unspecified
O73	Retained placenta and membranes, without haemorrhage
O73.0	Retained placenta without haemorrhage
O73.1	Retained portions of placenta and membranes, without haemorrhage
O75.4	Other complications of obstetric surgery and procedures
O75.8	Other specified complications of labour and delivery
O75.9	Complication of labour and delivery, unspecified
O87	Venous complications in the puerperium
O87.1	Deep phlebothrombosis in the puerperium
O87.3	Cerebral venous thrombosis in the puerperium
O87.9	Venous complication in the puerperium, unspecified
O88	Obstetric embolism
O88.0	Obstetric air embolism
O88.1	Amniotic fluid embolism
O88.2	Obstetric blood-clot embolism
O88.3	Obstetric pyaemic and septic embolism
O88.8	Other obstetric embolism
O90	Complications of the puerperium, not elsewhere classified
O90.0	Disruption of caesarean section wound
O90.1	Disruption of perineal obstetric wound
O90.2	Haematoma of obstetric wound
O90.3	Cardiomyopathy in the puerperium
O90.4	Postpartum acute renal failure
O90.5	Postpartum thyroiditis
O90.8	Other complications of the puerperium, not elsewhere classified

O90.9	Complication of the puerperium, unspecified
O29	Complications of anaesthesia during pregnancy
O29.0	Pulmonary complications of anaesthesia during pregnancy
O29.1	Cardiac complications of anaesthesia during pregnancy
O29.2	Central nervous system complications of anaesthesia during pregnancy
O29.3	Toxic reaction to local anaesthesia during pregnancy
O29.5	Other complications of spinal and epidural anaesthesia during pregnancy
O29.6	Failed or difficult intubation during pregnancy
O29.8	Other complications of anaesthesia during pregnancy
O29.9	Complication of anaesthesia during pregnancy, unspecified
O74	Complications of anaesthesia during labour and delivery
O74.0	Aspiration pneumonitis due to anaesthesia during labour and delivery
O74.1	Other pulmonary complications of anaesthesia during labour and delivery
O74.2	Cardiac complications of anaesthesia during labour and delivery
O74.3	Central nervous system complications of anaesthesia during labour and delivery
O74.4	Toxic reaction to local anaesthesia during labour and delivery
O74.6	Other complications of spinal and epidural anaesthesia during labour and delivery
O74.7	Failed or difficult intubation during labour and delivery
O74.8	Other complications of anaesthesia during labour and delivery
O74.9	Complication of anaesthesia during labour and delivery, unspecified
O89	Complications of anaesthesia during the puerperium
O89.0	Pulmonary complications of anaesthesia during the puerperium
O89.1	Cardiac complications of anaesthesia during the puerperium
O89.2	Central nervous system complications of anaesthesia during the puerperium
O89.3	Toxic reaction to local anaesthesia during the puerperium
O89.5	Other complications of spinal and epidural anaesthesia during the puerperium
O89.6	Failed or difficult intubation during the puerperium
O89.8	Other complications of anaesthesia during the puerperium
O89.9	Complication of anaesthesia during the puerperium, unspecified
O10	Pre-existing hypertension complicating pregnancy, childbirth and the puerperium
O10.0	Pre-existing essential hypertension complicating pregnancy, childbirth and the puerperium
O10.1	Pre-existing hypertensive heart disease complicating pregnancy, childbirth and the puerperium
O10.2	Pre-existing hypertensive renal disease complicating pregnancy, childbirth and the puerperium
O10.3	Pre-existing hypertensive heart and renal disease complicating pregnancy, childbirth and the puerperium
O10.4	Pre-existing secondary hypertension complicating pregnancy, childbirth and the puerperium
O10.9	Unspecified pre-existing hypertension complicating pregnancy, childbirth and the puerperium
O24	Diabetes mellitus in pregnancy
O24.0	Diabetes mellitus in pregnancy: Pre-existing diabetes mellitus, insulin-dependent
O24.1	Diabetes mellitus in pregnancy: Pre-existing diabetes mellitus, non-insulin-dependent
O24.2	Diabetes mellitus in pregnancy: Pre-existing malnutrition-related diabetes mellitus
O24.3	Diabetes mellitus in pregnancy: Pre-existing diabetes mellitus, unspecified
O24.9	Diabetes mellitus in pregnancy, unspecified
O98	Maternal infectious and parasitic diseases classifiable elsewhere but complicating pregnancy, childbirth and the puerperium
O98.0	Tuberculosis complicating pregnancy, childbirth and the puerperium
O98.1	Syphilis complicating pregnancy, childbirth and the puerperium
O98.2	Gonorrhoea complicating pregnancy, childbirth and the puerperium
O98.3	Other infections with a predominantly sexual mode of transmission complicating pregnancy, childbirth and the puerperium
O98.4	Viral hepatitis complicating pregnancy, childbirth and the puerperium
O98.5	Other viral diseases complicating pregnancy, childbirth and the puerperium
O98.6	Protozoal diseases complicating pregnancy, childbirth and the puerperium
O98.7	Human immunodeficiency [HIV] disease complicating pregnancy, childbirth and the puerperium
O98.8	Other maternal infectious and parasitic diseases complicating pregnancy, childbirth and the puerperium
O98.9	Unspecified maternal infectious or parasitic disease complicating pregnancy, childbirth and the puerperium
O99.0	Anaemia complicating pregnancy, childbirth and the puerperium
O99.1	Other diseases of the blood and blood-forming organs and certain disorders involving the immune mechanism complicating pregnancy, childbirth and the puerperium
O99.2	Endocrine, nutritional and metabolic diseases complicating pregnancy, childbirth and the puerperium
O99.3	Mental disorders and diseases of the nervous system complicating pregnancy, childbirth and the puerperium
O99.4	Diseases of the circulatory system complicating pregnancy, childbirth and the puerperium
O99.5	Diseases of the respiratory system complicating pregnancy, childbirth and the puerperium
O99.6	Diseases of the digestive system complicating pregnancy, childbirth and the puerperium
O99.7	Diseases of the skin and subcutaneous tissue complicating pregnancy, childbirth and the puerperium
O99.8	Other specified diseases and conditions complicating pregnancy, childbirth and the puerperium

O95	Obstetric death of unspecified cause
O96	Death from any obstetric cause occurring more than 42 days but less than one year after delivery
O96.0	Death from direct obstetric cause
O96.1	Death from indirect obstetric cause
O96.9	Death from unspecified obstetric cause
O97	Death from sequelae of direct obstetric causes
O97.0	Death from sequelae of direct obstetric cause
O97.1	Death from sequelae of indirect obstetric cause
O97.9	Death from sequelae of obstetric cause, unspecified
O08	Complications following abortion and ectopic and molar pregnancy
O08.0	Genital tract and pelvic infection following abortion and ectopic and molar pregnancy
O08.1	Delayed or excessive haemorrhage following abortion and ectopic and molar pregnancy
O08.2	Embolism following abortion and ectopic and molar pregnancy
O08.3	Shock following abortion and ectopic and molar pregnancy
O08.4	Renal failure following abortion and ectopic and molar pregnancy
O08.5	Metabolic disorders following abortion and ectopic and molar pregnancy
O08.6	Damage to pelvic organs and tissues following abortion and ectopic and molar pregnancy
O08.7	Other venous complications following abortion and ectopic and molar pregnancy
O08.8	Other complications following abortion and ectopic and molar pregnancy
O08.9	Complication following abortion and ectopic and molar pregnancy, unspecified
O21	Excessive vomiting in pregnancy
O21.0	Mild hyperemesis gravidarum
O21.8	Other vomiting complicating pregnancy
O21.9	Vomiting of pregnancy, unspecified
O22.0	Varicose veins of lower extremity in pregnancy
O22.1	Genital varices in pregnancy
O22.2	Superficial thrombophlebitis in pregnancy
O22.4	Haemorrhoids in pregnancy
O25	Malnutrition in pregnancy
O26	Maternal care for other conditions predominantly related to pregnancy
O26.0	Excessive weight gain in pregnancy
O26.1	Low weight gain in pregnancy
O26.2	Pregnancy care of habitual aborter
O26.3	Retained intrauterine contraceptive device in pregnancy
O26.4	Gestational pemphigoid
O26.5	Maternal hypotension syndrome
O26.7	Subluxation of symphysis (pubis) in pregnancy, childbirth and the puerperium
O26.8	Other specified pregnancy-related conditions
O26.9	Pregnancy-related condition, unspecified
O28	Abnormal findings on antenatal screening of mother
O28.0	Abnormal haematological finding on antenatal screening of mother
O28.1	Abnormal biochemical finding on antenatal screening of mother
O28.2	Abnormal cytological finding on antenatal screening of mother
O28.3	Abnormal ultrasonic finding on antenatal screening of mother
O28.4	Abnormal radiological finding on antenatal screening of mother
O28.5	Abnormal chromosomal and genetic finding on antenatal screening of mother
O28.8	Other abnormal findings on antenatal screening of mother
O28.9	Abnormal finding on antenatal screening of mother, unspecified
O29.4	Spinal and epidural anaesthesia-induced headache during pregnancy
O30	Multiple gestation
O30.0	Twin pregnancy
O30.1	Triplet pregnancy
O30.2	Quadruplet pregnancy
O30.8	Other multiple gestation
O30.9	Multiple gestation, unspecified
O30-O48	Maternal care related to the fetus and amniotic cavity and possible delivery problems
O31	Complications specific to multiple gestation
O31.0	Papyraceous fetus
O31.1	Continuing pregnancy after abortion of one fetus or more
O31.2	Continuing pregnancy after intrauterine death of one fetus or more
O31.8	Other complications specific to multiple gestation
O32	Maternal care for known or suspected malpresentation of fetus
O32.0	Maternal care for unstable lie

O32.1	Maternal care for breech presentation
O32.2	Maternal care for transverse and oblique lie
O32.3	Maternal care for face, brow and chin presentation
O32.4	Maternal care for high head at term
O32.5	Maternal care for multiple gestation with malpresentation of one fetus or more
O32.6	Maternal care for compound presentation
O32.8	Maternal care for other malpresentation of fetus
O32.9	Maternal care for malpresentation of fetus, unspecified
O33	Maternal care for known or suspected disproportion
O33.0	Maternal care for disproportion due to deformity of maternal pelvic bones
O33.1	Maternal care for disproportion due to generally contracted pelvis
O33.2	Maternal care for disproportion due to inlet contraction of pelvis
O33.3	Maternal care for disproportion due to outlet contraction of pelvis
O33.4	Maternal care for disproportion of mixed maternal and fetal origin
O33.5	Maternal care for disproportion due to unusually large fetus
O33.6	Maternal care for disproportion due to hydrocephalic fetus
O33.7	Maternal care for disproportion due to other fetal deformities
O33.8	Maternal care for disproportion of other origin
O33.9	Maternal care for disproportion, unspecified
O34	Maternal care for known or suspected abnormality of pelvic organs
O34.0	Maternal care for congenital malformation of uterus
O34.1	Maternal care for tumour of corpus uteri
O34.2	Maternal care due to uterine scar from previous surgery
O34.3	Maternal care for cervical incompetence
O34.4	Maternal care for other abnormalities of cervix
O34.5	Maternal care for other abnormalities of gravid uterus
O34.6	Maternal care for abnormality of vagina
O34.7	Maternal care for abnormality of vulva and perineum
O34.8	Maternal care for other abnormalities of pelvic organs
O34.9	Maternal care for abnormality of pelvic organ, unspecified
O35	Maternal care for known or suspected fetal abnormality and damage
O35.0	Maternal care for (suspected) central nervous system malformation in fetus
O35.1	Maternal care for (suspected) chromosomal abnormality in fetus
O35.2	Maternal care for (suspected) hereditary disease in fetus
O35.3	Maternal care for (suspected) damage to fetus from viral disease in mother
O35.4	Maternal care for (suspected) damage to fetus from alcohol
O35.5	Maternal care for (suspected) damage to fetus by drugs
O35.6	Maternal care for (suspected) damage to fetus by radiation
O35.7	Maternal care for (suspected) damage to fetus by other medical procedures
O35.8	Maternal care for other (suspected) fetal abnormality and damage
O35.9	Maternal care for (suspected) fetal abnormality and damage, unspecified
O36	Maternal care for other known or suspected fetal problems
O36.0	Maternal care for rhesus isoimmunization
O36.1	Maternal care for other isoimmunization
O36.2	Maternal care for hydrops fetalis
O36.3	Maternal care for signs of fetal hypoxia
O36.4	Maternal care for intrauterine death
O36.5	Maternal care for poor fetal growth
O36.6	Maternal care for excessive fetal growth
O36.7	Maternal care for viable fetus in abdominal pregnancy
O36.8	Maternal care for other specified fetal problems
O36.9	Maternal care for fetal problem, unspecified
O40	Polyhydramnios
O41	Other disorders of amniotic fluid and membranes
O41.0	Oligohydramnios
O41.8	Other specified disorders of amniotic fluid and membranes
O41.9	Disorder of amniotic fluid and membranes, unspecified
O42	Premature rupture of membranes
O42.0	Premature rupture of membranes, onset of labour within 24 hours
O42.1	Premature rupture of membranes, onset of labour after 24 hours
O42.2	Premature rupture of membranes, labour delayed by therapy
O42.9	Premature rupture of membranes, unspecified
O43.0	Placental transfusion syndromes

O43.1	Malformation of placenta
O43.8	Other placental disorders
O43.9	Placental disorder, unspecified
O47	False labour
O47.0	False labour before 37 completed weeks of gestation
O47.1	False labour at or after 37 completed weeks of gestation
O47.9	False labour, unspecified
O48	Prolonged pregnancy
O60	Preterm labour and delivery
O60.0	Preterm labour without delivery
O60.1	Preterm spontaneous labour with preterm delivery
O60.2	Preterm labour with term delivery
O60.3	Preterm delivery without spontaneous labour
O61	Failed induction of labour
O61.0	Failed medical induction of labour
O61.1	Failed instrumental induction of labour
O61.8	Other failed induction of labour
O61.9	Failed induction of labour, unspecified
O62	Abnormalities of forces of labour
O62.0	Primary inadequate contractions
O62.1	Secondary uterine inertia
O62.2	Other uterine inertia
O62.3	Precipitate labour
O62.4	Hypertonic, incoordinate, and prolonged uterine contractions
O62.8	Other abnormalities of forces of labour
O62.9	Abnormality of forces of labour, unspecified
O63	Long labour
O63.0	Prolonged first stage (of labour)
O63.1	Prolonged second stage (of labour)
O63.2	Delayed delivery of second twin, triplet, etc.
O63.9	Long labour, unspecified
O64	Obstructed labour due to malposition and malpresentation of fetus
O64.0	Obstructed labour due to incomplete rotation of fetal head
O64.1	Obstructed labour due to breech presentation
O64.2	Obstructed labour due to face presentation
O64.3	Obstructed labour due to brow presentation
O64.4	Obstructed labour due to shoulder presentation
O64.5	Obstructed labour due to compound presentation
O64.8	Obstructed labour due to other malposition and malpresentation
O64.9	Obstructed labour due to malposition and malpresentation, unspecified
O65	Obstructed labour due to maternal pelvic abnormality
O65.0	Obstructed labour due to deformed pelvis
O65.1	Obstructed labour due to generally contracted pelvis
O65.2	Obstructed labour due to pelvic inlet contraction
O65.3	Obstructed labour due to pelvic outlet and mid-cavity contraction
O65.4	Obstructed labour due to fetopelvic disproportion, unspecified
O65.5	Obstructed labour due to abnormality of maternal pelvic organs
O65.8	Obstructed labour due to other maternal pelvic abnormalities
O65.9	Obstructed labour due to maternal pelvic abnormality, unspecified
O66	Other obstructed labour
O66.0	Obstructed labour due to shoulder dystocia
O66.1	Obstructed labour due to locked twins
O66.2	Obstructed labour due to unusually large fetus
O66.3	Obstructed labour due to other abnormalities of fetus
O66.4	Failed trial of labour, unspecified
O66.5	Failed application of vacuum extractor and forceps, unspecified
O66.8	Other specified obstructed labour
O66.9	Obstructed labour, unspecified
O68	Labour and delivery complicated by fetal stress [distress]
O68.0	Labour and delivery complicated by fetal heart rate anomaly
O68.1	Labour and delivery complicated by meconium in amniotic fluid
O68.2	Labour and delivery complicated by fetal heart rate anomaly with meconium in amniotic fluid
O68.3	Labour and delivery complicated by biochemical evidence of fetal stress

O68.8	Labour and delivery complicated by other evidence of fetal stress
O68.9	Labour and delivery complicated by fetal stress, unspecified
O69	Labour and delivery complicated by umbilical cord complications
O69.0	Labour and delivery complicated by prolapse of cord
O69.1	Labour and delivery complicated by cord around neck, with compression
O69.2	Labour and delivery complicated by other cord entanglement, with compression
O69.3	Labour and delivery complicated by short cord
O69.4	Labour and delivery complicated by vasa praevia
O69.5	Labour and delivery complicated by vascular lesion of cord
O69.8	Labour and delivery complicated by other cord complications
O69.9	Labour and delivery complicated by cord complication, unspecified
O70	Perineal laceration during delivery
O70.0	First degree perineal laceration during delivery
O70.1	Second degree perineal laceration during delivery
O70.2	Third degree perineal laceration during delivery
O70.3	Fourth degree perineal laceration during delivery
O70.9	Perineal laceration during delivery, unspecified
O74.5	Spinal and epidural anaesthesia-induced headache during labour and delivery
O75	Other complications of labour and delivery, not elsewhere classified
O75.0	Maternal distress during labour and delivery
O75.1	Shock during or following labour and delivery
O75.2	Pyrexia during labour, not elsewhere classified
O75.5	Delayed delivery after artificial rupture of membranes
O75.6	Delayed delivery after spontaneous or unspecified rupture of membranes
O75.7	Vaginal delivery following previous caesarean section
O80	Single spontaneous delivery
O80.0	Spontaneous vertex delivery
O80.1	Spontaneous breech delivery
O80.8	Other single spontaneous delivery
O80.9	Single spontaneous delivery, unspecified
O80-O84	Delivery
O81	Single delivery by forceps and vacuum extractor
O81.0	Low forceps delivery
O81.1	Mid-cavity forceps delivery
O81.2	Mid-cavity forceps with rotation
O81.3	Other and unspecified forceps delivery
O81.4	Vacuum extractor delivery
O81.5	Delivery by combination of forceps and vacuum extractor
O82	Single delivery by caesarean section
O82.0	Delivery by elective caesarean section
O82.1	Delivery by emergency caesarean section
O82.2	Delivery by caesarean hysterectomy
O82.8	Other single delivery by caesarean section
O82.9	Delivery by caesarean section, unspecified
O83	Other assisted single delivery
O83.0	Breech extraction
O83.1	Other assisted breech delivery
O83.2	Other manipulation-assisted delivery
O83.3	Delivery of viable fetus in abdominal pregnancy
O83.4	Destructive operation for delivery
O83.8	Other specified assisted single delivery
O83.9	Assisted single delivery, unspecified
O84	Multiple delivery
O84.0	Multiple delivery, all spontaneous
O84.1	Multiple delivery, all by forceps and vacuum extractor
O84.2	Multiple delivery, all by caesarean section
O84.8	Other multiple delivery
O84.9	Multiple delivery, unspecified
O87.0	Superficial thrombophlebitis in the puerperium
O87.2	Haemorrhoids in the puerperium
O87.8	Other venous complications in the puerperium
O89.4	Spinal and epidural anaesthesia-induced headache during the puerperium
O92	Other disorders of breast and lactation associated with childbirth

O92.0	Retracted nipple associated with childbirth
O92.1	Cracked nipple associated with childbirth
O92.2	Other and unspecified disorders of breast associated with childbirth
O92.3	Agalactia
O92.4	Hypogalactia
O92.5	Suppressed lactation
O92.6	Galactorrhoea
O92.7	Other and unspecified disorders of lactation
O94	Sequelae of complication of pregnancy, childbirth and the puerperium
O64	Obstructed labour due to malposition and malpresentation of fetus
O64.0	Obstructed labour due to incomplete rotation of fetal head
O64.1	Obstructed labour due to breech presentation
O64.2	Obstructed labour due to face presentation
O64.3	Obstructed labour due to brow presentation
O64.4	Obstructed labour due to shoulder presentation
O64.5	Obstructed labour due to compound presentation
O64.8	Obstructed labour due to other malposition and malpresentation
O64.9	Obstructed labour due to malposition and malpresentation, unspecified
O65	Obstructed labour due to maternal pelvic abnormality
O65.0	Obstructed labour due to deformed pelvis
O65.1	Obstructed labour due to generally contracted pelvis
O65.2	Obstructed labour due to pelvic inlet contraction
O65.3	Obstructed labour due to pelvic outlet and mid-cavity contraction
O65.4	Obstructed labour due to fetopelvic disproportion, unspecified
O65.5	Obstructed labour due to abnormality of maternal pelvic organs
O65.8	Obstructed labour due to other maternal pelvic abnormalities
O65.9	Obstructed labour due to maternal pelvic abnormality, unspecified
O66	Other obstructed labour
O66.0	Obstructed labour due to shoulder dystocia
O66.1	Obstructed labour due to locked twins
O66.2	Obstructed labour due to unusually large fetus
O66.3	Obstructed labour due to other abnormalities of fetus
O66.8	Other specified obstructed labour
O66.9	Obstructed labour, unspecified
O64	Obstructed labour due to malposition and malpresentation of fetus
O64.0	Obstructed labour due to incomplete rotation of fetal head
O64.1	Obstructed labour due to breech presentation
O64.2	Obstructed labour due to face presentation
O64.3	Obstructed labour due to brow presentation
O64.4	Obstructed labour due to shoulder presentation
O64.5	Obstructed labour due to compound presentation
O64.8	Obstructed labour due to other malposition and malpresentation
O64.9	Obstructed labour due to malposition and malpresentation, unspecified
O65	Obstructed labour due to maternal pelvic abnormality
O65.0	Obstructed labour due to deformed pelvis
O65.1	Obstructed labour due to generally contracted pelvis
O65.2	Obstructed labour due to pelvic inlet contraction
O65.3	Obstructed labour due to pelvic outlet and mid-cavity contraction
O65.4	Obstructed labour due to fetopelvic disproportion, unspecified
O65.5	Obstructed labour due to abnormality of maternal pelvic organs
O65.8	Obstructed labour due to other maternal pelvic abnormalities
O65.9	Obstructed labour due to maternal pelvic abnormality, unspecified
O66	Other obstructed labour
O66.0	Obstructed labour due to shoulder dystocia
O66.1	Obstructed labour due to locked twins
O66.2	Obstructed labour due to unusually large fetus
O66.3	Obstructed labour due to other abnormalities of fetus
O66.8	Other specified obstructed labour
O66.9	Obstructed labour, unspecified

Table S2: MMR by background characteristics in Bangladesh in 2016, presented in deaths per 100,000 live births with 95% confidence interval

		MMR	Lower interval	Upper interval
Residence	Rural	204	165	243
	Urban	147	91	203
Age in years	15- 24 years	150	110	190
	25- 29 years	161	103	219
	30+ years	326	231	420
Education	No education	351	203	499
	Primary (<=5 years)	250	180	320
	Secondary or more (6+ years)	135	100	170
Wealth quintile	Poor	234	177	290
	Middle	197	122	273
	Rich	138	94	183
Overall	Maternal Mortality Ratio	196	159	234
